# Supplementary material for: A Comprehensive Molecular and Clinical Investigation of Approved Anti-HCV Drugs Repurposing against SARS-CoV-2 Infection: A Glaring Gap between Benchside and Bedside Medicine
Source: Vaccines (Basel). 2023 Feb 22;11(3):515. doi: 10.3390/vaccines11030515 (PMC10056950; doi:10.3390/vaccines11030515)
Supplement: Supplementary file 1 [file vaccines-11-00515-s001.zip › Supplementary Information S2.pdf]

RdRp-SOF and –remdesivir molecular interaction and simulation

The binding pockets residues like Asp623, Thr680, Thr687 and Asp760 were involved in polar contact formation with remdesivir. Pi-interactions of Arg553, Asp623, Arg624 and Ser681 with a functional group of ligands stabilized the ligand binding (Figure S1A). Similarly, SOF formed multiple H-bonds with Thr556, Asp-623, Arg-624 and Ser-628. Besides this, Cys622 was involved in Pi-sulfur interaction with aromatic groups and Asp623 formed attractive interaction with a charged group of the ligand (Figure S1B). RdRp, Remdesivir and SOF showed RMSD in range of 0.1 to 0.4 nm (Figure S1C and D).

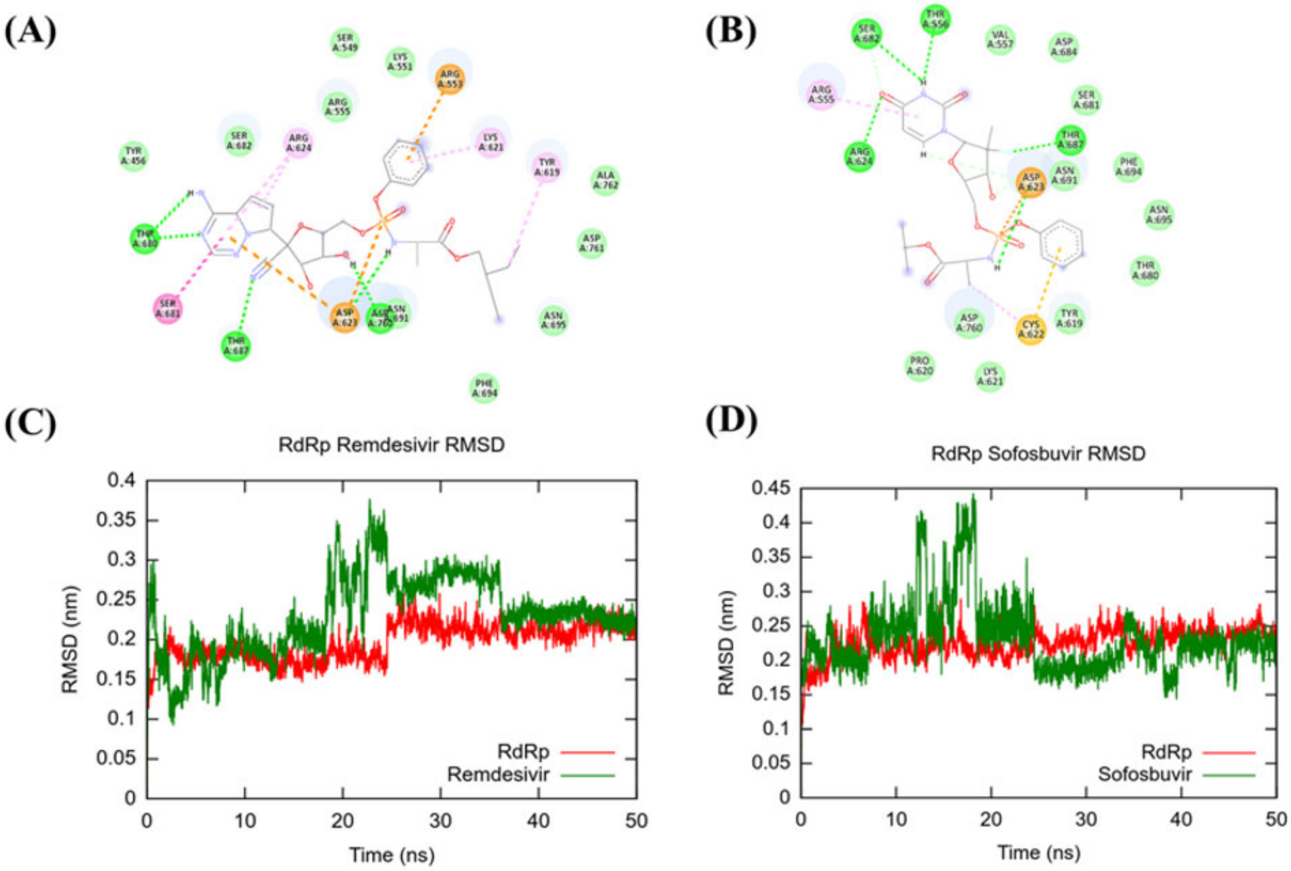

Figure S1. Molecular interactions of RdRp with (**A**) Remdesivir (**B**) SOF. RMSD of RdRp in complex with (**C**) Remdesivir (**D**) SOF upon 50ns similar.

Potential energy plots for simulated complexes

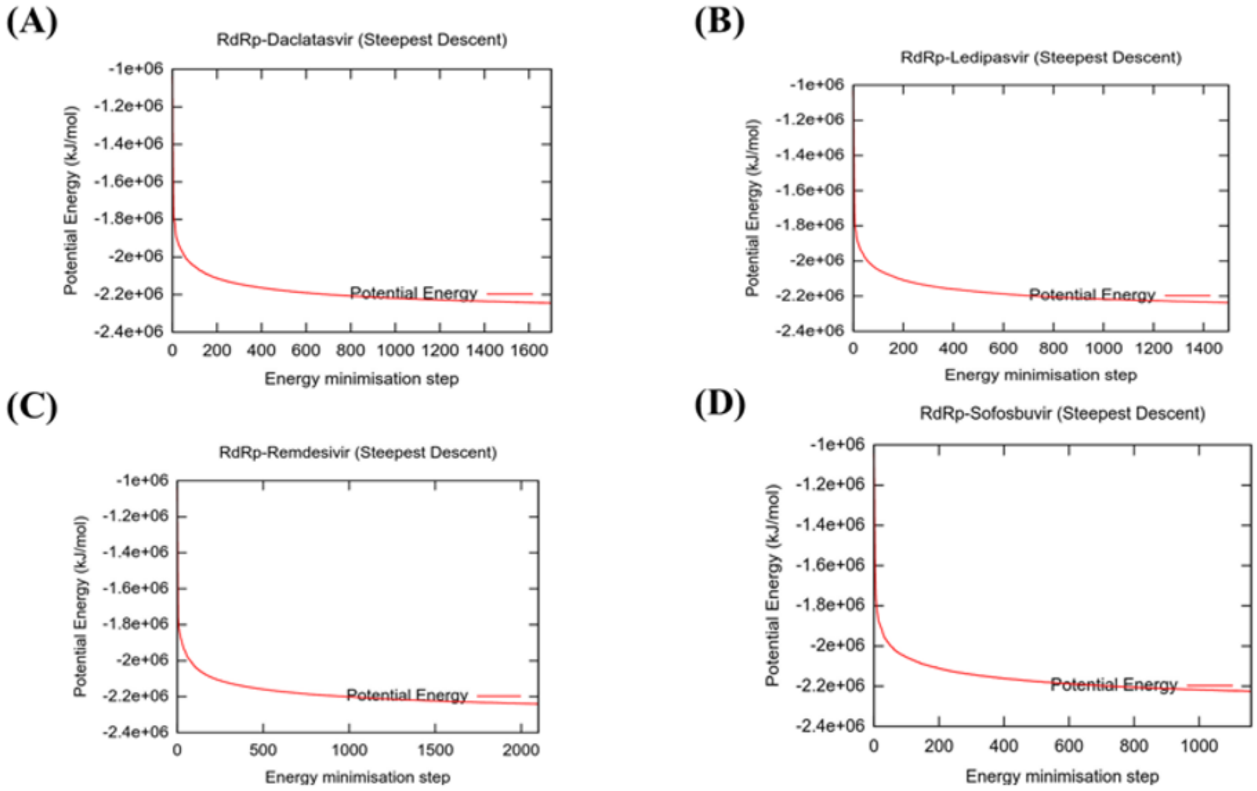

Figure S2. Potential energy plots for RdRp in complex with (**A**) DCV (**B**) LDP (**C**) Remdesivir and (**D**) SOF after 50 ns simulation.

Table S1. Binding score and predicted affinity of selected ligands against RdRp of SARS-CoV-2.

Ligand	Binding Score (Kcal/mol)	Binding Affinty (−log10(KD|Ki))
Remdesvir	−8.2	11.6
Ledipasvir	−9.4	93.6
Daclatasvir	−7.8	24.3
Sofosbuvir	−7.6	−25.4
